# PP47 Analysis Of Technical-Scientific Reports Of Judicialized Health Technologies From The e-NATJus System: Cross-Sectional Study

**DOI:** 10.1017/S0266462325102663

**Published:** 2025-12-29

**Authors:** Isabela Porto De Toledo, Ana Luiza Cabrera Martimbianco, Camila Monteiro Cruz, Carolina de Oliveira Cruz Latorraca, Cecília Menezes Farinasso, Patrícia do Carmo Silva Parreira, Rafael Leite Pacheco, Roberta Borges Silva, Verônica Colpani, Rachel Riera

## Abstract

**Introduction:**

Brazil’s Unified Health System (SUS) ensures universal and free healthcare access. However, when technologies are unavailable in this system, users can appeal to the courts. To support such decisions, the National Council of Justice established technical support centers that provide technical-scientific reports (TRs) to evaluate health technologies. This study aimed to analyze these TRs and rank the most judicialized health conditions.

**Methods:**

This descriptive cross-sectional study was conducted at the Center of Health Technology Assessment, Hospital Sírio-Libanês, São Paulo-SP, Brazil, using the TRs available in the e-NATJus system (https://www.pje.jus.br/e-natjus/pesquisaPublica.php). The sample was limited to TRs published between 3 December 2018 and 12 November 2024. Quantitative analyses were summarized as percentages.

**Results:**

Over 235,000 TRs were analyzed, with requests rising from 1,004 in 2019 to 71,120 in 2024. Medications represented 56.1 percent of TR demands, and 56.5 percent of the requested technologies were available in SUS. Favorable rulings occurred in 50.5 percent of cases. The most judicialized conditions were autism spectrum disorder (ASD), insulin-dependent diabetes, non-insulin-dependent diabetes, prostate cancer, and diabetic retinopathy. The main requests for ASD were aripiprazole, cannabis derivates, and applied behavior analysis therapy. Insulin glargine was the most requested drug for insulin-dependent diabetes, dapagliflozin for non-insulin-dependent diabetes, abiraterone acetate for prostate cancer, and ranibizumab for diabetic retinopathy.

**Conclusions:**

The surge in TRs highlights growing demand for innovative treatments, many of which are unavailable in the SUS or lack robust evidence. Access challenges persist even for included therapies, emphasizing the need for equitable access and reliable evidence to guide decisions.

